# Editorial: Purinergic signaling in metabolic diseases and inflammation pharmacology

**DOI:** 10.3389/fphar.2025.1631388

**Published:** 2025-06-19

**Authors:** Alejandro Sánchez-Melgar, Juana Maria Sanz, Anna Lisa Giuliani

**Affiliations:** ^1^ Department of Inorganic, Organic Chemistry and Biochemistry, Faculty of Chemical Sciences and Technologies, Institute of Biomedicine (IB-UCLM), Instituto de Investigación Sanitaria de Castilla-La Mancha (IDISCAM), University of Castilla-La Mancha, Ciudad Real, Spain; ^2^ Department of Chemistry and Pharmaceutical Science, University of Ferrara, Ferrara, Italy; ^3^ Department of Medical Sciences, University of Ferrara, Ferrara, Italy

**Keywords:** inflammation, purinergic signalling, metabolic disorders, aging, drugs

## Introduction

Numerous diseases, including obesity, diabetes, cardiovascular disorders, dementia-associated cognitive decline and sarcopenia are characterized by underlying inflammatory processes that contribute to disease progression. Purinergic signalling is widely recognized as a key modulator of inflammation. This system involves extracellular nucleotides (i.e., ATP, ADP, UTP, and UDP) and nucleosides (i.e., adenosine), which are detected by two receptor families: P2 receptors (for nucleotides) and P1 receptors (for nucleosides), expressed on various cell types. The regulation of extracellular ATP and adenosine levels is mediated by ecto-enzymes, particularly NTPDase1/CD39 and 5′-Nucleotidase/CD73, which sequentially degrade ATP to adenosine, as well as by nucleotide/nucleoside transporters. Generally, ATP-activated P2 receptors promote pro-inflammatory responses, whereas adenosine, acting via P1 receptors, exerts predominantly anti-inflammatory and immunosuppressive effects. Consequently, modulating inflammatory conditions through purinergic signalling may represent a promising therapeutic strategy for these diseases.

In this Research Topic we have compiled six original research articles that provide up-to-date insights into the role of purinergic signalling in different inflammatory diseases.

## Purinergic signalling in metabolic disorders

Metabolic disorders represent a major cause of morbidity and mortality in developed countries. These disorders are often associated to obesity that is closely linked to chronic low-grade inflammation, primarily originating from dysfunctional adipose tissue. As adipose tissue expands, adipocytes become hypertrophic, leading to mechanical and metabolic stress that promotes the release of inflammatory mediators such as tumor necrosis factor-alpha (TNF-α), interleukin-6 (IL-6), interleukin-1alpha (IL-1α) and monocyte chemoattractant protein-1 (MCP-1) These signals attract immune cells, particularly macrophages, that infiltrate the adipose tissue and shift toward a pro-inflammatory M1 phenotype (Kawai et al., 2021). The immune activation further amplifies inflammation through secretion of additional cytokines, production of reactive oxygen species (ROS), and activation of transcription factors like NF-κB. The inflammatory milieu disrupts insulin signalling pathways, contributing to insulin resistance and metabolic syndrome, thereby linking inflammation in obesity to a broad range of systemic dysfunctions (Hotamisligil, 2006).

The low-grade inflammation that accompanies obesity very likely implies the activation of P2X7R, a main receptor for extracellular ATP (eATP) (Adinolfi et al., 2018), contributing to adipose tissue dysfunction. Among the regulatory mechanisms of P2X7R expression, the role of sex steroids remains unclear. Authors described that under non-inflammatory (basal) conditions, treatment of human adipocytes with either testosterone (T) or 17β-estradiol (E2) did not significantly alter the P2X7R gene expression (Di Vincenzo et al.). In contrast, exposure to dihydrotestosterone (DHT), a non-aromatizable androgen, led to a notable downregulation of P2X7R gene expression. Exposure of adipocytes to LPS, significantly increased P2X7R expression compared to baseline levels. Remarkably, pre-treatment with DHT prior to LPS exposure further enhanced the P2X7R gene expression, indicating that DHT sensitizes adipocytes to inflammatory activation. A similar enhancement was observed when adipocytes were pre-treated with testosterone in combination with anastrozole, an aromatase inhibitor that prevents conversion of testosterone into estradiol. While this combination had no significant effect on P2X7R expression at rest, it led to a pronounced upregulation following LPS stimulation. These findings highlight the modulatory role of sex steroids, particularly non-aromatizable androgens like DHT, on purinergic signaling in adipocytes. The results suggest that androgens can exert dual effects depending on the inflammatory state of the tissue and point to a sex-dependent regulation of adipose tissue inflammation via the P2X7R, a key component of the immune response machinery.

Obesity can contribute to inflammatory evolution in different tissues and organs. Supernatants from the periprostatic adipose tissue (PPAT) of obese mice showed significantly elevated levels of total nitrate and nitrite (NOx^−^) and adenosine compared to controls, and diminished phenylephrine (PE)-induced contractions, but only in prostate tissues from obese mice (Passos et al.). This anticontractile effect was mitigated when the PPAT was co-incubated with NO pathway inhibitors or adenosine receptor (AR) antagonists, suggesting these pathways as mediators of the observed effects. PPAT supernatants from obese animals also enhanced the viability of PNT1-A normal human epithelial cells and suppressed caspase-3 expression, indicating anti-apoptotic activity. Moreover, BPH-1 (hyperplastic human epithelial) cells exhibited increased proliferation at all time points when exposed to PPAT supernatant, an effect significantly blunted by blocking NO signaling or AR activation. These findings highlight a functional role for NO and adenosine released from PPAT in modulating prostate contractility and epithelial cell proliferation. In obesity, this paracrine interaction may contribute to prostate dysfunction and disease progression. Further studies are warranted to explore the therapeutic potential of targeting PPAT-derived NO and adenosine in prostate-related pathologies.

An established model for obesity-induced type 2 diabetes, represented by Zucker Diabetic Fatty (ZDF) rats, was employed to investigate the impact of Cannabidiol (CBD), a non-psychoactive compound derived from the cannabis plant with a wide range of reported therapeutic properties, on the adenosinergic signalling pathway within the myocardium, specifically focused on the left atria (Viczjan et al.). The animals, fed with a diabetogenic diet, received daily oral administration of CBD (60 mg/kg) or vehicle for 4 weeks. The functional effects were assessed using two AR agonists: CPA, a selective A1R agonist, and adenosine itself, which is rapidly metabolized and transported inside the myocardial cells. The response to CPA was significantly reduced in CBD-treated rats compared to controls, suggesting a potential desensitization or receptor downregulation. In contrast, adenosine elicited a markedly stronger effect in the CBD group, except at the highest concentrations tested. This differential response supports the hypothesis that CBD inhibits adenosine transport, leading to increased extracellular adenosine accumulation and enhanced receptor activation, rather than acting as a direct receptor agonist. Chronic CBD administration may therefore potentiate adenosine-mediated cardio protection by sustaining elevated interstitial adenosine levels. However, the possibility of partial A1R downregulation, due to prolonged adenosine exposure, cannot be ruled out, which might have tempered the full extent of the observed activation. This study provides the first direct functional evidence that CBD can inhibit adenosine reuptake in the myocardium, suggesting a novel mechanism through which CBD may exert cardiovascular benefits, particularly under diabetic conditions.

## Purinergic signalling in pathogens-induced neuroinflammation and neurodegeneration

Infections can trigger systemic inflammation and activate immune responses in the brain, leading to neuroinflammation. This process involves the release of pro-inflammatory mediators that can stimulate endothelial cells from the blood-brain barrier (BBB) allowing activation of microglia and astrocytes, which can promote oxidative stress and neuroinflammation (Acioglu and Elkabes, 2025). Chronic or severe neuroinflammation contributes to synaptic loss and neuronal damage, increasing the risk of long-term cognitive impairment and neurodegenerative diseases (McManus and Heneka, 2017). An overwhelming inflammatory reaction that adversely affects the brain can result as a complication of pathogen induced inflammatory response. A dysregulated immune response to infection is represented by sepsis, a complex, life-threatening condition, whose complication at the central nervous system (CNS) is known as sepsis-associated encephalopathy. This neuroinflammatory response, triggered by pathogen recognition, leads to cellular stress and the release of ATP, which activates the P2X7R, that is highly expressed in the CNS. Even though the P2X7R has been implicated in various chronic neurodegenerative and neuroinflammatory conditions, its specific role in long-term neurological outcomes following sepsis remains poorly understood. Therefore, a recent study (Alves et al.) has evaluated neuroinflammatory and behavioural changes in surviving wild-type (WT), P2X7 knockout (P2X7^−/−^), or Brilliant Blue G (BBG)-treated mice after sepsis induced by caecal ligation and perforation (CLP). On day 13 post-surgery, various parameters were evaluated: cognitive performance, assessed through novel object recognition and Water T-maze tests, acetylcholinesterase activity, glial activation markers (Iba-1 and GFAP), and levels of inflammatory cytokines. Following sepsis, both WT and P2X7^−/−^ mice exhibited memory impairments, but deletion of P2X7R partially mitigated the increase in AChE activity in the cortex and reduced glial activation, as indicated by lower Iba-1 and GFAP expression in the cerebral cortex. Notably, GFAP elevation occurred in the cortex but not in the hippocampus of both genotypes. Furthermore, P2X7R deletion or inhibition by BBG led to decreased production of pro- and anti-inflammatory cytokines, including IL-1β, TNF-α, and IL-10. These findings suggest that targeting the P2X7R could help reduce neuroinflammation and cognitive dysfunction in sepsis survivors, positioning it as a promising therapeutic target for sepsis-associated encephalopathy (Alves et al.).

Leprosy is a chronic infectious disease caused by *Mycobacterium leprae*, which can lead to debilitating neurodegenerative complications. The *M. leprae* primarily infects skin macrophages and Schwann cells, the glial cells of the peripheral nervous system, where it alters host lipid metabolism to support its own survival by promoting the formation of cholesterol-rich lipid droplets (LDs), which serve as nutrient reservoirs and a protective niche. Although the understanding of leprosy pathogenesis has improved, the molecular mechanisms underlying host-pathogen interactions, particularly those involving neuroimmune signalling, remain uncompletely understood. The purinergic system, which relies on eATP and adenosine as key signalling molecules, plays essential roles in immune regulation, lipid metabolism, and neuron-glia communication. To date, the role in leprosy of the adenosine A2A receptor (A2AR), that is of particular interest due to its known involvement in inflammation and neurodegenerative diseases, has not been fully investigated. A recent study (Dos Santos et al.) has shown that *M. leprae* infection upregulated the expression of two enzymes involved in adenosine metabolism, CD73 and adenosine deaminase (ADA), while downregulated A2AR expression and levels of phosphorylated CREB (p-CREB), a key transcription factor in A2AR signalling. Notably, these effects were reversed by pharmacological activation of A2AR with the selective agonist CGS21680 which reduced LDs accumulation, expression of lipogenic genes, production of the pro-inflammatory cytokines IL-6 and IL-8, and the intracellular viability of *M. leprae*, while p-CREB levels were increased. These findings suggest that *M. leprae* suppresses A2AR-mediated pathways in Schwann cells, thereby facilitating LDs formation and bacterial persistence. The study highlights the potential of targeting A2AR signalling as a therapeutic strategy to limit *M. leprae* survival and mitigate nerve damage in leprosy, suggesting an unrecognized role of the purinergic system in the neuropathogenesis of this disease.

## Purinergic signalling in age-related disorders

Sarcopenia, the progressive loss of muscle mass and strength, is closely linked to various age-related conditions such as bone frailty, osteoporosis, type 2 diabetes (Antonioli et al., 2015), and cardiovascular diseases (Dalle et al., 2017; Kalyani et al., 2014). As muscle function declines, mobility and metabolic health are impaired, increasing the risk of falls, disability, and hospitalization in older adults. Sarcopenia often coexists with chronic inflammation and hormonal changes, further accelerating the progression of age-associated pathologies. Proper ATP balance is essential during muscle differentiation (myogenesis), and the purinergic system plays a key role in this process. How adenosine signalling influences myogenesis has been recently investigated in a C2C12 myoblast cell line by using tenofovir, an inhibitor of ATP release, and dipyridamole, which blocks cellular uptake of adenosine (Marco-Bonilla et al.). Tenofovir treatment increased intracellular ATP, and reduced extracellular adenosine, thus resulting in decreased Pax7 and prematurely elevated MHC expression, indicating accelerated differentiation. In contrast, dipyridamole elevated intracellular AMP and extracellular adenosine levels, reversing the effects of tenofovir and promoting a more regulated myogenic progression. All four subtypes of adenosine receptors were present throughout the differentiation process, with dipyridamole notably enhancing the expression of the A2BR. Tenofovir inhibited AMPK activation and led to decreased levels of cAMP, PKAα, and p-CREB; however, these effects were reversed upon treatment with dipyridamole. Both ATP and adenosine likely serve as critical modulators of muscle differentiation. Inhibiting ATP release by tenofovir leads to premature myogenesis, while dipyridamole, restoring a balanced differentiation process through A2BR-induced cAMP/AMPK pathway, may represent a promising therapeutic strategy in sarcopenia.

In conclusion, different aspects of inflammation related to metabolic diseases have been illustrated in the cited studies which underlie the central role of purinergic signalling. Therapeutic strategies for these pathologies may therefore envisage the use of drugs that modulate purinergic pathways.
